# Monitoring of Venous and Arterial Occlusion With Remote Interstitial Tissue Glucose Measurement Systems in a Rabbit Free Flap Model

**DOI:** 10.1002/micr.70214

**Published:** 2026-03-15

**Authors:** Canberk M. Gurbuz, Ceyhun Uzun, Oguzhan Eroglu, Emrah K. Yasar, Murat S. Alagoz

**Affiliations:** ^1^ Kocaeli University Hospital Kocaeli Turkey

## Abstract

**Background:**

Free tissue transfer requires meticulous postoperative monitoring to detect vascular occlusion. Although experimental studies have explored the relationship between interstitial glucose levels and tissue perfusion, long‐term evaluation of glucose levels during and after occlusion‐reperfusion has not been thoroughly investigated. The study objective was to investigate the correlation between controlled venous and arterial occlusion and changes in interstitial tissue glucose, using a remote interstitial glucose monitoring device.

**Materials and Methods:**

This experimental study was conducted on eight New Zealand White rabbits, each weighing between 3.2 and 3.8 kg, under general anesthesia, utilizing a 4 × 8 cm perforator flap supplied by skin perforators originating from the thoracodorsal artery. Interstitial glucose levels within the flaps were continuously monitored using FreeStyle Libre flash glucose monitoring system. Baseline glucose levels were recorded 1 day prior to vascular occlusion, followed by monitoring between 15 min intervals during experimental clamping of both venous ischemia (75 min), venous decongestion (75 min), and arterial occlusion (45 min). Criteria for detecting vessel occlusion were established based on changes in interstitial glucose concentration.

**Results:**

Venous occlusion was associated with a significant increase in interstitial glucose levels. At 15 min post‐occlusion, interstitial glucose increased by 47.8%, which was significantly higher than baseline (*p = 0.018*). However, at 30 min post‐unclamping, interstitial glucose declined by 18.3% (*p* = 0.028) and by 57.4% over 75 min. In contrast, arterial occlusion was associated with a rapid decline in glucose levels. At 15 min post‐occlusion, interstitial glucose decreased by 56% (*p = 0.018*). Total necrosis was observed in all flaps followed by arterial occlusion.

**Conclusions:**

Interstitial glucose monitoring appears to be a reliable method for detecting vascular occlusion in free tissue transfers within this experimental model. This technique may offer a rapid, minimally invasive, and cost‐effective approach for postoperative vascular monitoring of free flaps. Further investigation in human trials is warranted to confirm these findings and assess clinical utility.

## 1 Introduction

Although free flap surgery is widely performed, flap monitoring protocols vary considerably among plastic surgery units. Previous studies have demonstrated that flap failure predominantly occurs within the first 72 h postoperatively, and the likelihood of successful salvage is closely associated with the timing of vascular compromise (Chen et al. 2007). The mean time to intervention for salvaged free flaps was 30.8 h, compared to 51.5 h for flaps that were not salvaged. Notably, approximately 71% of compromised flaps required reoperation within the first 48 h, highlighting the critical importance of intensive monitoring during this early postoperative period. The risk of flap compromise decreases progressively after the third day and remains relatively constant through postoperative day seven and beyond (Shen et al. 2021).

A variety of techniques have been described for monitoring free flaps. These include clinical assessment of flap color and turgor, capillary refill, temperature, and advanced technologies such as laser Doppler flowmetry (Yuen and Feng 2000), color duplex sonography (Yano et al. 2003), implantable Doppler systems (Swartz et al. 1994), near‐infrared spectroscopy (NIRS) (Irwin et al. 1995), microdialysis (Setala et al. 2006; Udesen et al. 2000), and tissue pH monitoring (Dunn et al. 1993). The most commonly used strategy combines external Doppler monitoring with routine clinical evaluation. However, determining the optimal duration and frequency of such monitoring can be challenging, especially in settings where nursing staff may have limited experience with flap physiology. Moreover, prolonged and frequent monitoring can be uncomfortable and disruptive for patients (Devine et al. 2001).

An ideal monitoring modality should be non‐invasive, safe, accurate, reliable, and capable of rapidly detecting vascular changes. It should also be user‐friendly, reproducible, cost‐effective, and portable. Direct monitoring of tissue metabolites has shown that interstitial glucose levels respond rapidly to perfusion changes (Rojdmark et al. 1998). Although microdialysis provides valuable metabolic insights, its complexity limits clinical application. In contrast, transcutaneous glucose monitoring devices, originally developed for diabetic patients, have rapidly advanced in accessibility and practicality (Sun et al. 2024).

While clinical studies have explored the relationship between interstitial glucose levels and flap monitoring, obtaining a sufficient sample size is challenging due to low flap failure rates (Nemoto et al. 2025). Therefore, experimental studies have been conducted to further investigate this relationship (Sitzman et al. 2010). Building upon this existing research, we designed an experimental model to examine the relationship between interstitial glucose levels and experimentally induced arterial and venous occlusion, allowing for the observation of reperfusion values and long‐term follow‐up.

We hypothesize that continuous interstitial glucose monitoring would offer a rapid and accurate means of detecting vascular occlusion in free tissue transfer. This study employed a rabbit free flap model to assess interstitial glucose dynamics following first venous and subsequent arterial occlusion, as well as during reperfusion.

## 2 Materials and Methods

All procedures were approved by the Kocaeli University Animal Care and Use Institute Commission. Eight adult New Zealand White rabbits, each weighing between 3.2 and 3.8 kg, were acclimated for 3 days prior to the operation.

### 2.1 Flap Model

**FIGURE 1:**
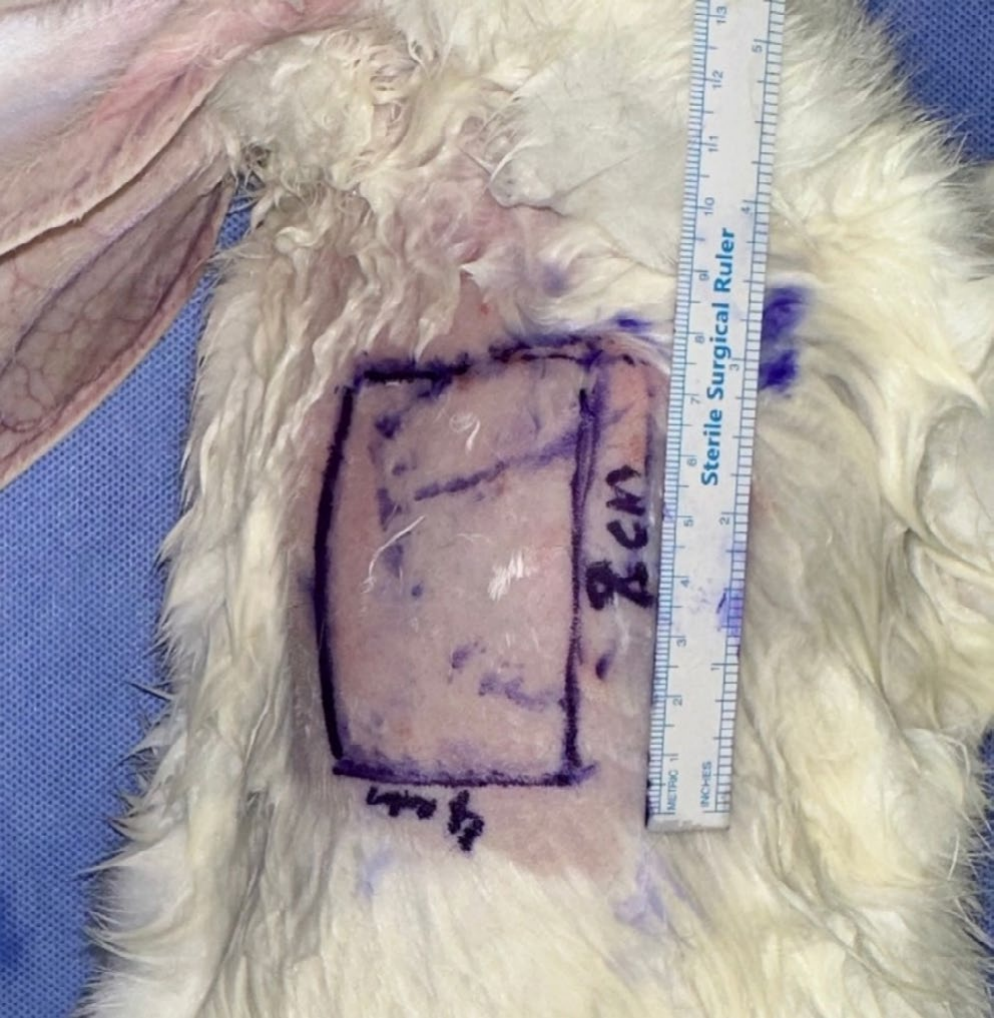
Surgical planning of the thoracodorsal artery‐based perforator flap. After palpation of the scapula (dotted line), a 4 × 8 cm flap was planned to be located caudolateral to the scapula.

**FIGURE 2:**
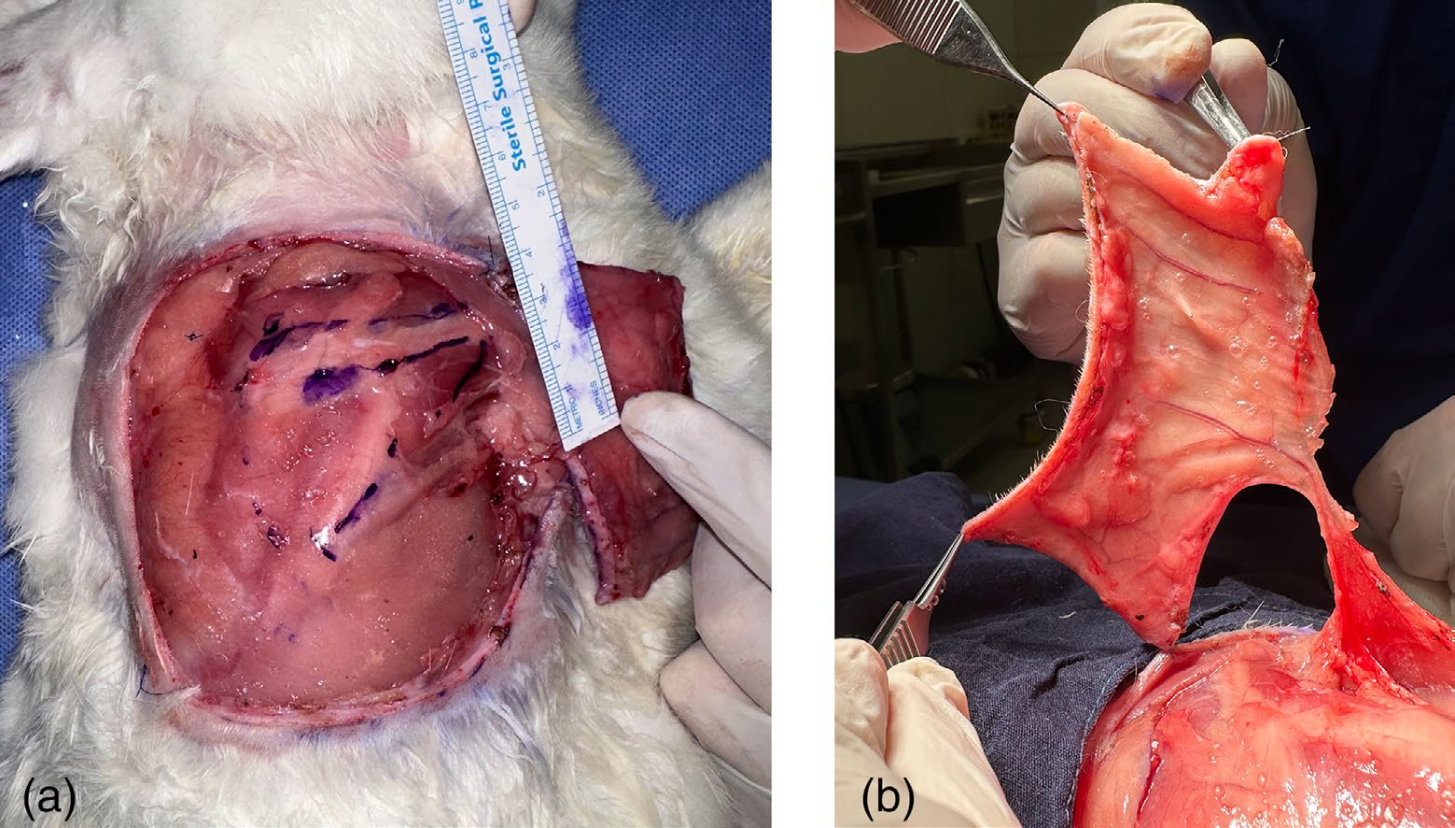
(A) The S1 perforator flap pedicle localization and pedicle length are seen. In our dissections, the flap localization was seen to be quite stable. The pedicle courses to the axillary region 2 cm below the spine of the scapula. (B) The frequent anastomoses between the vessels seen in the flap allow the flap to be dissected in large sizes.

**FIGURE 3:**
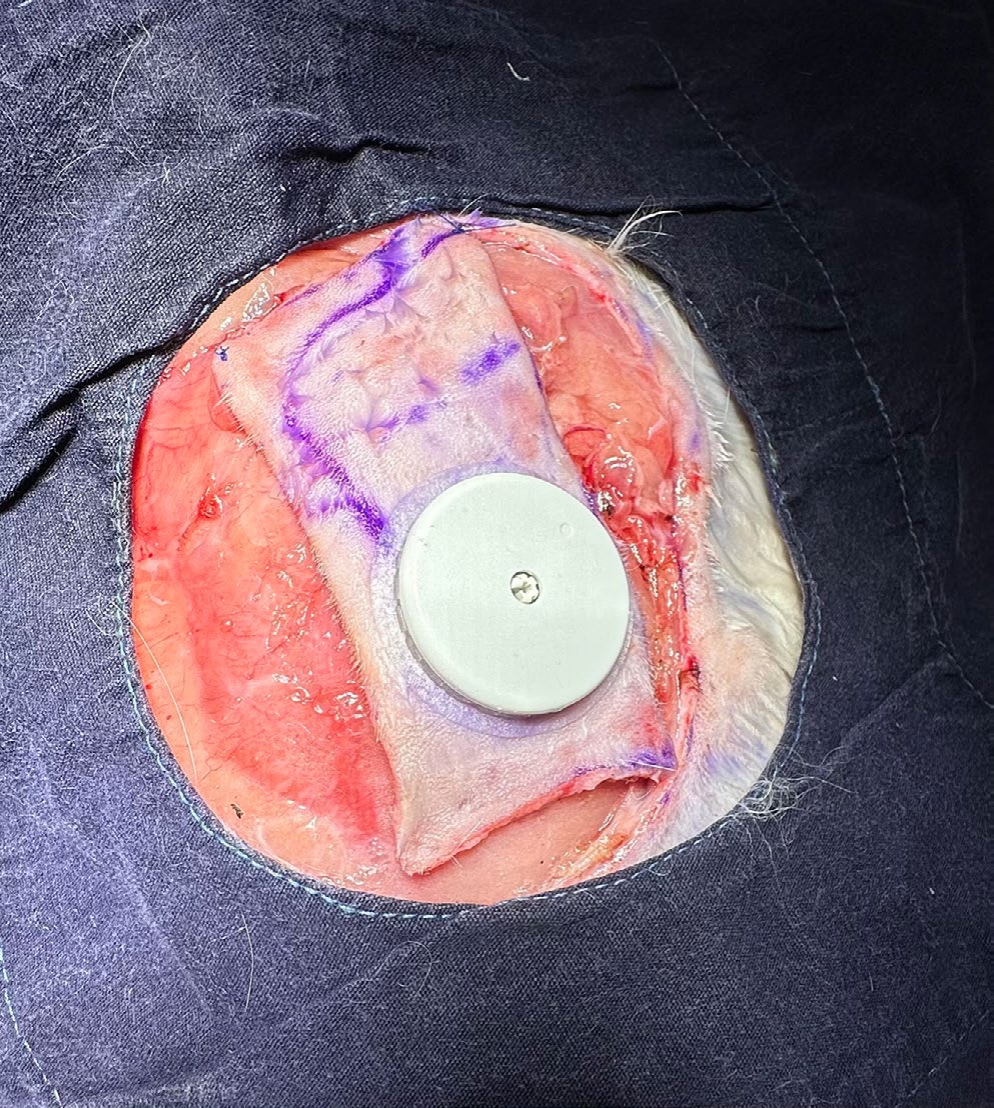
In each subject, the glucometer was placed in the distal half of the planned flap.

On the first day of the procedure, under appropriate anesthesia, The FreeStyle Libre interstitial glucose measurement probe was applied to the cutaneous surface of the planned flap. 4 × 8 cm island flap was carefully dissected, with its blood supply originating from skin perforators of the thoracodorsal artery in the caudolateral region of the scapula (Figure 1). The flap also included the panniculus carnosus tissue. During the incision of the medial, inferior, and superior edges of the flap, all visible vascular structures beneath the flap were preserved to ensure adequate blood flow. Following the incision along the lateral edge, the vascular pedicle was traced proximally toward the subscapular vessels until an adequate length was achieved. This technique resulted in a cutaneous flap with a vascular pedicle approximately 2 cm in length (Figure 2). The interstitial glucose measurement probe was applied to the center of the distal half of the cutaneous surface of the planned flap (Figure 3).

The vascular anatomy of the rabbit dorsal flank was consistent, and the harvest of the latissimus dorsi musculocutaneous (LDMC) flap was reliable. Subsequent studies in rabbits identified a muscle‐perforating branch supplying the dorsal skin (Angel et al. 1990). A large skin flap could be raised independently of the latissimus dorsi muscle based on this perforator alone. The first perforator branch (S1) was observed to pierce the proximal portion of the latissimus dorsi muscle and the dorsal thoracic fascia of the rabbit. In our dissections, this branch was consistently located 2 cm below the scapular spine, tracing toward the axilla (Figure 2). The viability of the flap was initially assessed through physical examination and further confirmed using Doppler flow measurement.

### 2.2 Experimental Design

**FIGURE 4:**
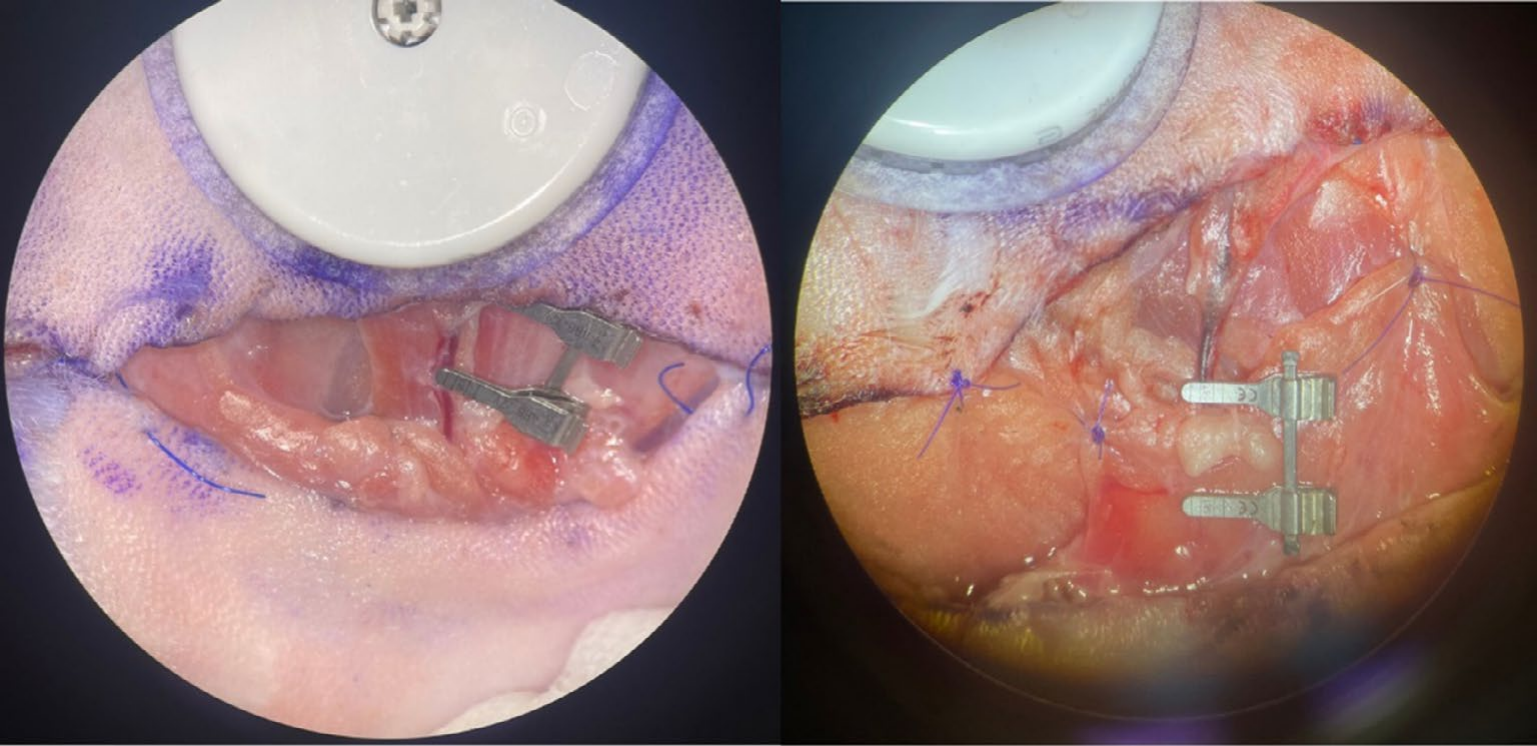
Microscopic image of congestion distal to the clamp after venous clamping.

After monitoring glucose levels and flap viability for 24 h, the subject was anesthetized again. Following induction of anesthesia, only the lateral portion of the flap was desutured, and baseline glucose monitoring was conducted for 1 h. After this, the S1 perforator vein was clamped for 75 min, and interstitial glucose measurements were recorded at 15‐min intervals. Venous congestion was confirmed through microscopic inspection and Doppler ultrasonography (Figure 4). Afterward, the clamp was released, restoring circulation, and glucose measurements were recorded every 15 min for another 75 min. The lateral side of the flap was resutured, and the subject was awakened, continuing with glucose measurements and viability monitoring.

**FIGURE 5:**
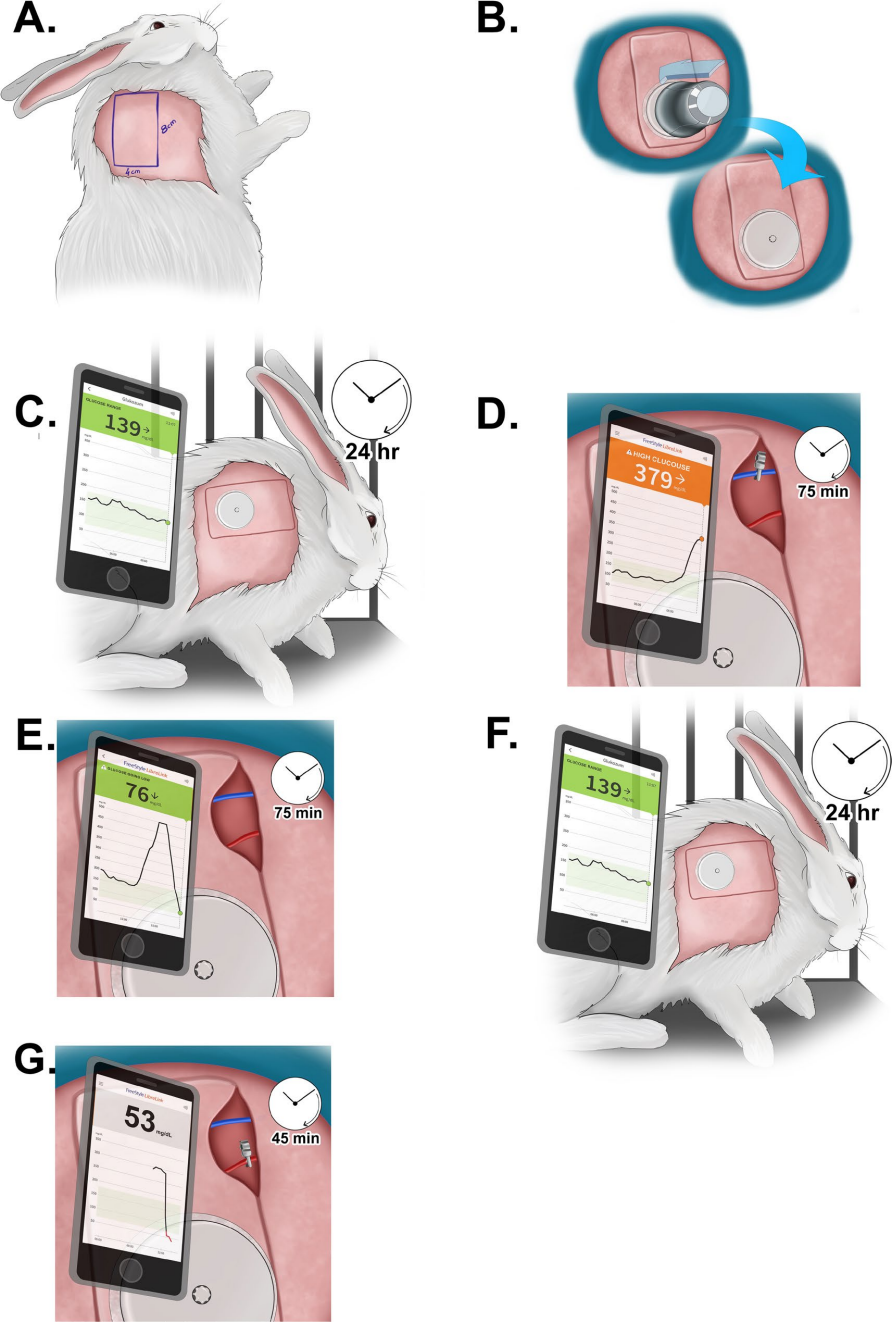
A 4 × 8 cm island flap was elevated (A), and an interstitial glucose probe was placed on the distal half of the flap (B). After 24 h of baseline monitoring (C), the animals were re‐anesthetized, the lateral flap edge was reopened, and 1 h of baseline glucose measurements were obtained. Venous occlusion of the S1 perforator vein was then induced for 75 min (D), followed by 75 min of reperfusion monitoring (E). The flap was resutured and postoperative monitoring continued. After an additional 24 h (F), the S1 perforator artery on the contralateral side was occluded for 45 min (G), with glucose levels recorded at 15‐min intervals. The clamp was released, the flap was repositioned, and routine monitoring resumed.

Twenty‐four hours after venous congestion, the S1 perforator‐based fasciocutaneous flap was elevated on the contralateral side. Baseline glucose changes were noted for 1 h using the glucometer probe adapted to the flap. Subsequently, the S1 perforator artery was occluded with a clamp, and glucose measurements were recorded at 15‐min intervals for 45 min. After 45 min, the clamp was released, and the flap was repositioned. Once the subject was awakened, glucose measurements and flap viability monitoring resumed (Figure 5).

### 2.3 Statistical Analyses

All statistical analyses were conducted using IBM SPSS Statistics version 29.0 (IBM Corp., Armonk, NY, USA). Normality was assessed with the Shapiro–Wilk test. As assumptions for normal distribution were not met, non‐parametric tests were employed. The Mann–Whitney U test was used for independent comparisons, and the Wilcoxon signed‐rank test for paired comparisons. Bonferroni correction was considered for multiple comparisons to control Type I error risk. A *p*‐value < 0.05 was considered statistically significant.

## 3 Results

Eight animals underwent elevation of bilateral S1 perforator flaps with selective venous and arterial occlusion in two flaps per animal. One animal was excluded from the study due to the lack of viability in the distal portion of the flap after elevation.

### 3.1 Venous Occlusion

**FIGURE 6:**
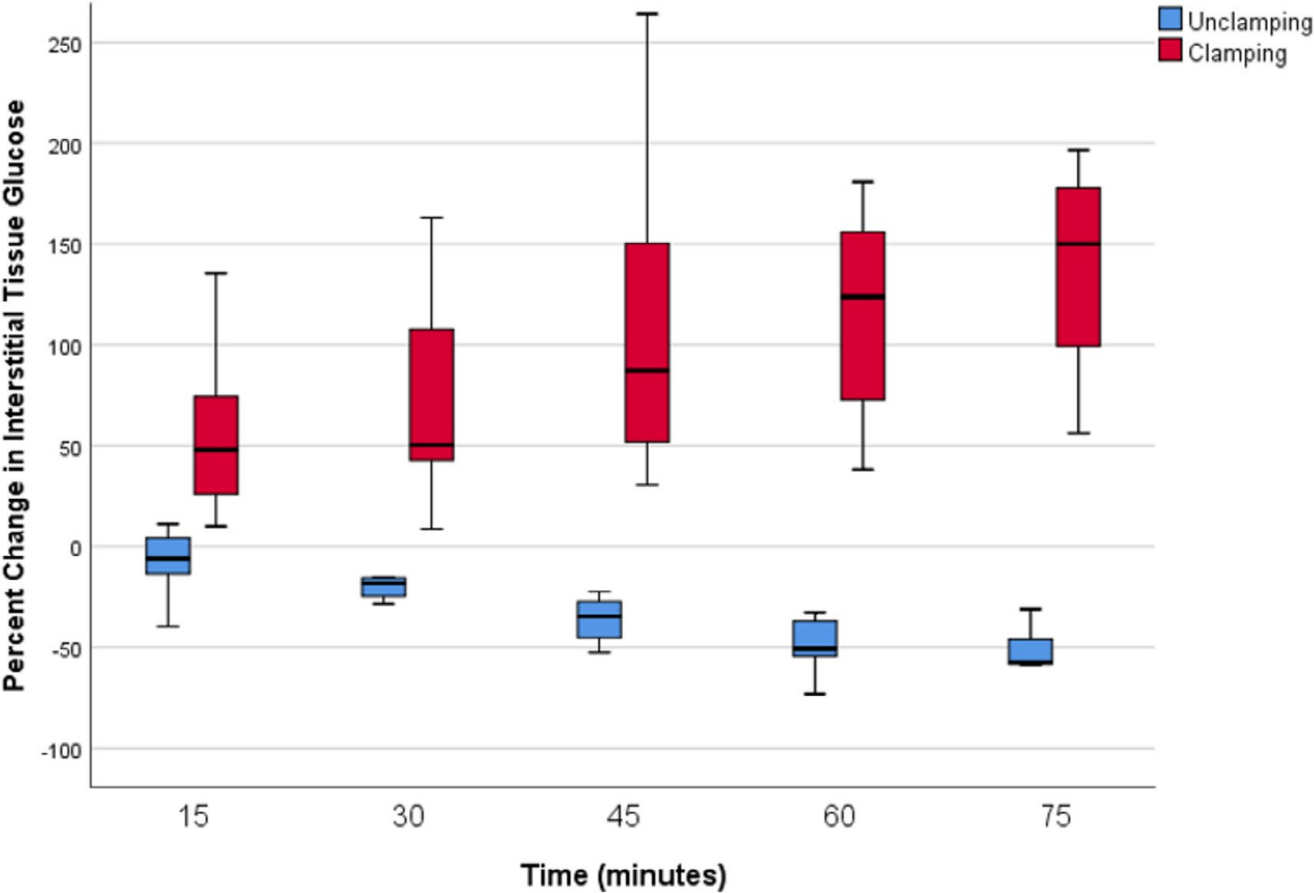
Boxplot graph of median percentage change in the interstitial glucose values after venous clamping and subsequent unclamping. As seen in the graph, an average median glucose increase of 47.8% was found at the 15th minute after clamping. The first significant value after venous unclamping was obtained at the 30th minute with an average value of 18.3%.

Prior to venous occlusion, the interstitial glucose levels over time in the S1 perforator flap of each animal were 144 mg/dL (anesthesia induction), 143 mg/dL (30 min after induction), and 156 mg/dL (60 min after induction) (*p =* 0.459). Following venous occlusion, interstitial glucose levels increased over time in the occluded flap. At 15 min after venous occlusion, the median interstitial glucose in the occluded flap was significantly higher than the baseline value (156 vs. 223 mg/dL; *p* = 0.018). Interstitial glucose increased by 47.8% in the first 15 min and by 150% over 75 min (Figure 6, Table 1).

**TABLE 1:** Summary of key interstitial glucose level comparisons across different timepoints during venous occlusion.

Venous	Median (IQR)
Baseline	210 (84–239)
15 min	261 (167–370)
30 min	352 (221–388)
45 min	375 (264–440)
60 min	382 (311–450)
75 min	401 (236–457)

Multiple comparisons	*p* ^a^
Baseline versus 15 min	0.018
Baseline versus 30 min	0.018
Baseline versus 45 min	0.018
Baseline versus 60 min	0.018
Baseline versus 75 min	0.028

Abbreviation: IQR, interquartile range.

^a^ Wilcoxon signed‐rank test was used for time point comparisons.

### 3.2 Venous Decongestion

After venous unclamping, interstitial glucose levels decreased over time in the flap. At 15 min after venous unclamping, interstitial glucose in the flap was lower than in the occluded flap; the difference was not significant (404 vs. 334 mg/dL; *p* = 0.11). Interstitial glucose decreased by an average of 6% in the first 15 min following venous unclamping. At 30 min after venous unclamping, interstitial glucose in the flap was significantly lower than in the occluded flap (404 vs. 289 mg/dL; *p* = 0.028) and, following venous unclamping, interstitial glucose decreased by 18.3% in the first 30 min and by 57.4% over 75 min (Figure 6, Table 2).

**TABLE 2:** Summary of key interstitial glucose level comparisons across different timepoints during venous unclamping.

	Median (IQR)
Venous 75	404 (245–450)
Unclamping 15	334 (265–467)
Unclamping 30	289 (208–418)
Unclamping 45	240 (160–337)
Unclamping 60	199 (121–223)
Unclamping 75	172 (103–205)

Multiple comparisons	*p* ^a^
Venous 75 versus Unclamping 15	0.310
Venous 75 versus Unclamping 30	0.028
Venous 75 versus Unclamping 45	0.028
Venous 75 versus Unclamping 60	0.018
Venous 75 versus Unclamping 75	0.018

Abbreviation: IQR, interquartile range.

^a^ Wilcoxon signed‐rank test was used for time point comparisons.

### 3.3 Arterial Occlusion

**FIGURE 7:**
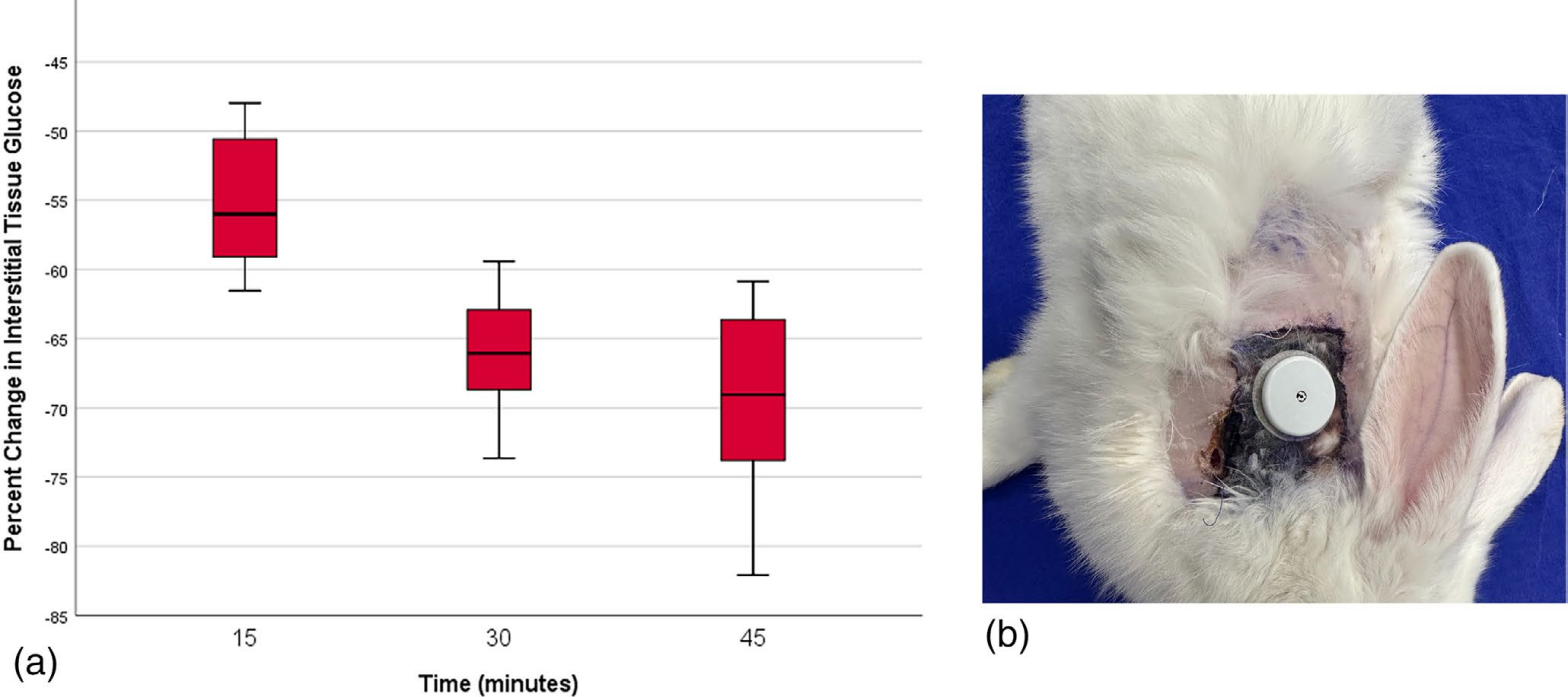
(A) After arterial occlusion, a rapid decrease in glucose was observed in the first 15 min with a median decrease of 56%. (B) Non‐viable flap tissue was observed in the late follow‐up after irreversible arterial occlusion; necrotic tissue was debrided and the skin was primarily repaired.

Following arterial occlusion, interstitial glucose in the flap rapidly decreased over time. At 15 min after arterial clamping, median interstitial glucose in the flap was significantly lower than the baseline value (168 vs. 77 mg/dL; *p* = 0.018). After arterial clamping, interstitial glucose declined by a mean of 56% in the first 15 min and by 69% over 45 min (Table 3). The glucometer issued a “not detected” warning for all animals with glucose values below 50 mg/dL, and glucose measurements could not be recorded after the clamp was released, as the device ceased measurements following repeated “not detected” warnings. Flap viability follow‐up demonstrated that arterial occlusion was irreversible, with all flaps becoming necrotic (Figure 7).

**TABLE 3:** Summary of key interstitial glucose level comparisons across different timepoints during arterial occlusion.

Arterial	Median (IQR)
Baseline	168 (143–235)
15 min	77 (55–112)
30 min	56 (54–72)
45 min	52 (52–54)

Multiple comparisons	*p* ^a^
Baseline versus 15 min	0.018
Baseline versus 30 min	0.018
Baseline versus 45 min	0.018

Abbreviation: IQR, interquartile range.

^a^ Wilcoxon signed‐rank test was used for time point comparisons.

## 4 Discussion

Venous obstruction remains a major cause of both partial and complete failure of free flaps. Acute venous obstruction is recognized as a more critical threat to flap survival than arterial occlusion or combined arterial and venous occlusion (Angel et al. 1990) (Heden and Sollevi 1989). Optimal clinical outcomes are achieved when venous compromise is identified and addressed early (Hidalgo and Jones 1990). The ideal monitoring system should feature continuous tracking of quantifiable tissue data, rapid and accurate detection of ischemia, be noninvasive or minimally invasive, readily available, and cost‐effective (De Block et al. 2008). Blood glucose measurement systems, widely used for monitoring blood glucose in patients with diabetes, possess these features. Previous research has highlighted the potential of glucose monitoring in assessing flap viability, which prompted the current experimental study (Perez Colman et al. 2023). The present study suggests that interstitial glucose monitoring using a transcutaneous monitor has the potential to meet many of the criteria for an ideal flap monitoring system.

The literature reports an overall free flap failure rate of approximately 5.8%, with the majority of flap losses occurring within the first 72 h (%79,6) postoperatively and frequently requiring surgical re‐exploration (Shen et al. 2021). Although intermittent clinical evaluation and external Doppler monitoring are widely used in routine practice, there is currently no clear consensus regarding the optimal duration or modality of postoperative flap surveillance. The 2‐week continuous monitoring period flash glucose monitoring system covers the timeframe during which a significant percentage of flap failures are reported to occur.

**FIGURE 8:**
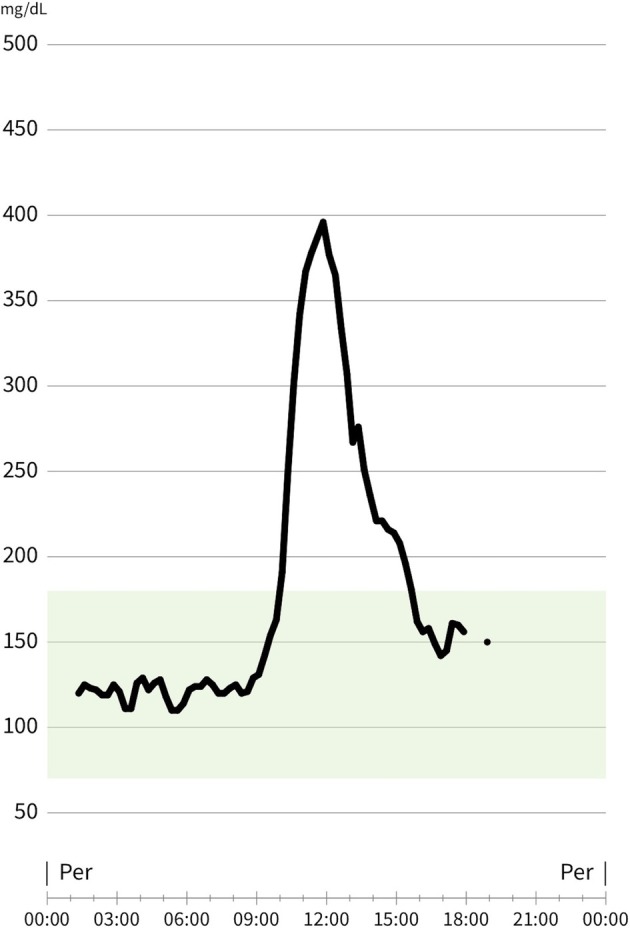
Screenshot of interstitial tissue glucose monitoring graph taken from mobile application before venous clamping and clamp release. Glucose change for 8 h before measurement was retained in application memory. Thus, it was observed that glucose change values of rabbits fed with *ad libitum* were very low during feeding compared to venous occlusion.

The rabbit model was chosen for this study due to its thicker subcutaneous tissue compared to rats, larger surface area, and blood glucose range that is more comparable to that of humans (Silva et al. 2020). Probe stabilization remained consistent even after the animal was awakened, allowing for long‐term follow‐up. This long‐term monitoring allowed for the observation of systemic factors that could influence glucose levels; however, these factors did not produce changes in interstitial tissue glucose of the same magnitude as those observed during venous or arterial congestion (Figure 8).

Another advantage of the rabbit model was the consistent location of the vascular pedicle. In this study, the rabbit S1 perforator flap, which is supplied by skin perforators originating from the thoracodorsal artery in the caudolateral region of the scapula and includes the panniculus carnosus tissue, was used (Angel et al. 1990; Guerra et al. 2005). In our experience, it was observed that the vascular pedicle, consisting of one artery and one vein, consistently originated 2 cm below the spine of the scapula and extended distally toward the axillary region in each dissection (Figure 2). Although no additional tests were performed to objectively assess flap perfusion, the consistent anatomy of the S1 perforator likely mitigated this limitation. The glucometer, positioned centrally in the distal half of the flap, showed similar baseline values in all animals.

**FIGURE 9:**
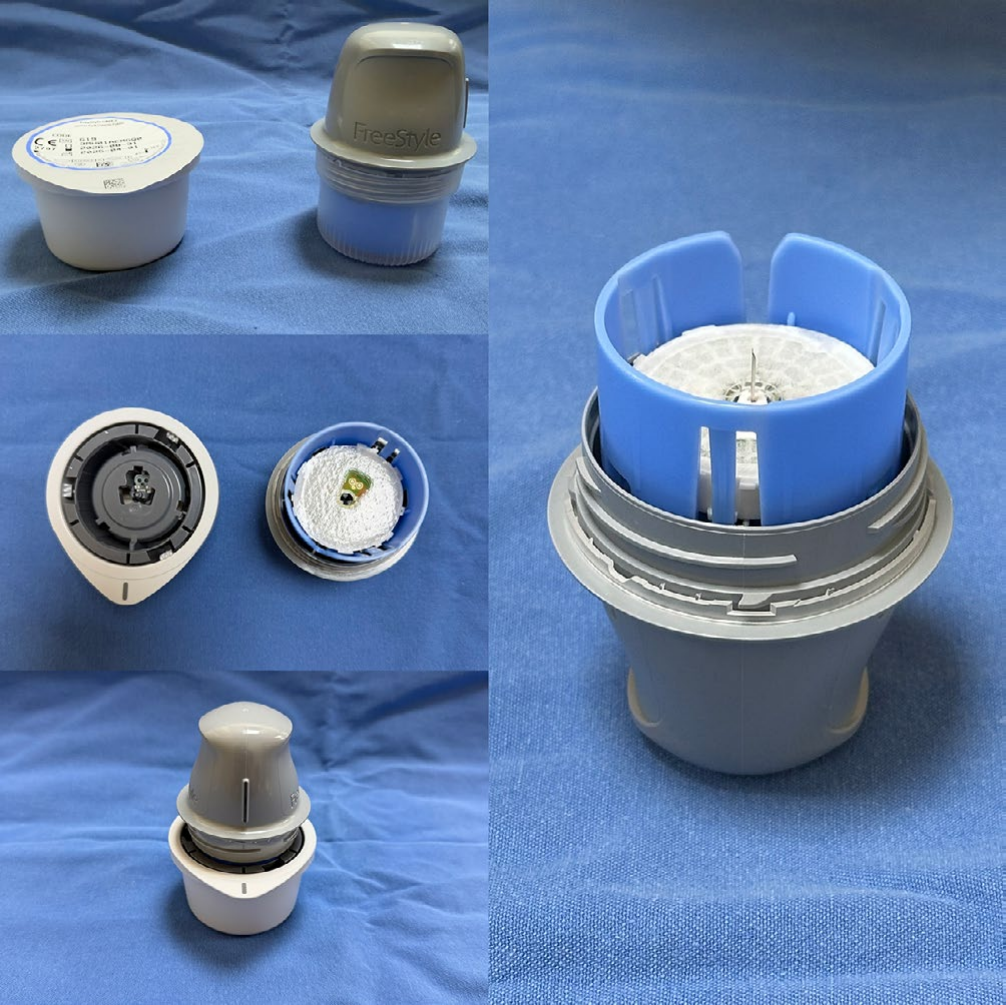
To prepare the sensor, first remove the lid from the Sensor Pack entirely. Detach the cap from the Sensor Applicator and align the dark indicator marks on both the applicator and the pack. Place the pack on a firm surface and press the applicator straight down until it stops. Then lift the applicator out of the pack. Position it over the selected application site and press firmly to insert and secure the sensor.

The FreeStyle Libre flash glucose monitoring system (Abbott Diabetes Care Inc. Witney, United Kingdom) measures interstitial glucose levels. The system consists of a sensor and a reader, with an optional companion app for mobile devices. In this study, we used the FreeStyle Libre mobile phone application to simulate clinical practice. The sensor is supplied with a sensor pack and sensor applicator (Figure 9). The sensor, 35 mm in diameter, is designed for a 14‐day placement. It contains a thin (0.4 mm), flexible, and sterile fiber that is inserted into the skin to a depth of 5 mm. The fiber draws interstitial fluid into the sensor, where glucose levels are automatically measured every minute and stored at 15‐min intervals for 8 h. Glucose levels can be viewed at any time by scanning the reader over the sensor. To scan, the reader is held 1–4 cm above the sensor for 1 s, displaying current glucose levels, levels over the previous 8 h, and trends in glucose levels (whether increasing or decreasing, and at what rate) (Blum 2018).

**FIGURE 10:**
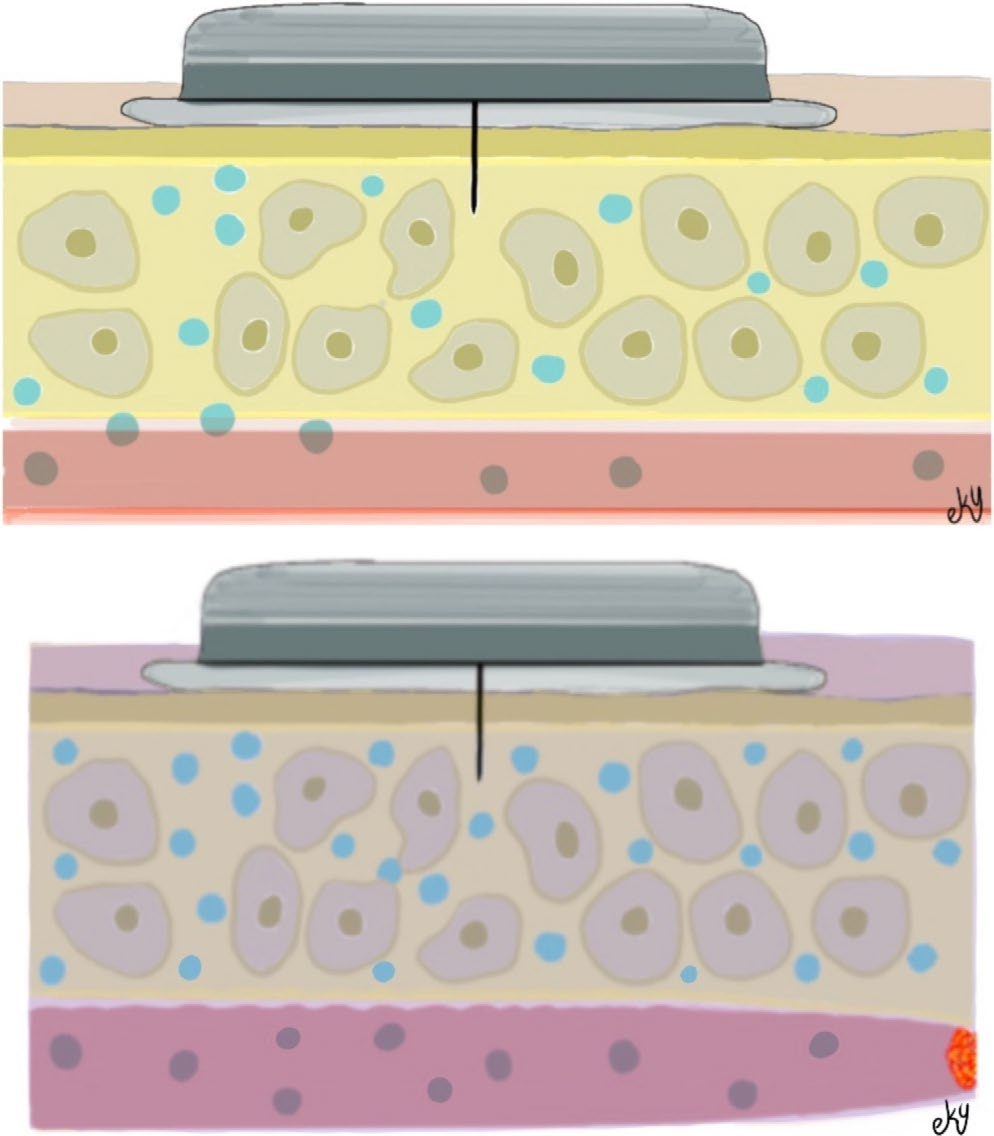
Illustration of glucose leakage into the interstitial tissue due to endothelial damage following venous occlusion.

The detrimental effects of flap venostasis include edema, which hinders interstitial oxygen diffusion and causes external vascular compression, as well as endothelial cell damage, which triggers inflammatory mediators and may ultimately result in thrombosis (Hjortdal et al. 1992; Marzella et al. 1988). Electron microscopy of flaps with venous occlusion has demonstrated the extravasation of white blood cells, as well as the presence of activated platelets, fibrin, and red blood cells within the distended and disrupted capillaries (Hjortdal et al. 1994). Our hypothesis posited that capillary damage would lead to the leakage of glucose from the vessel into the interstitial tissue, resulting in an increase in glucose concentration due to compression in the interstitial fluid (Figure 10). While earlier studies have reported a decrease in interstitial glucose with venous occlusion (Sitzman et al. 2010), our findings indicated an increase. This discrepancy may be attributable to differences in the experimental occlusion models used. Future experimental studies may clarify this situation. Our results suggest that a 47.8% increase in flap interstitial glucose may be an indicator of venous ischemia in this model.

**FIGURE 11:**
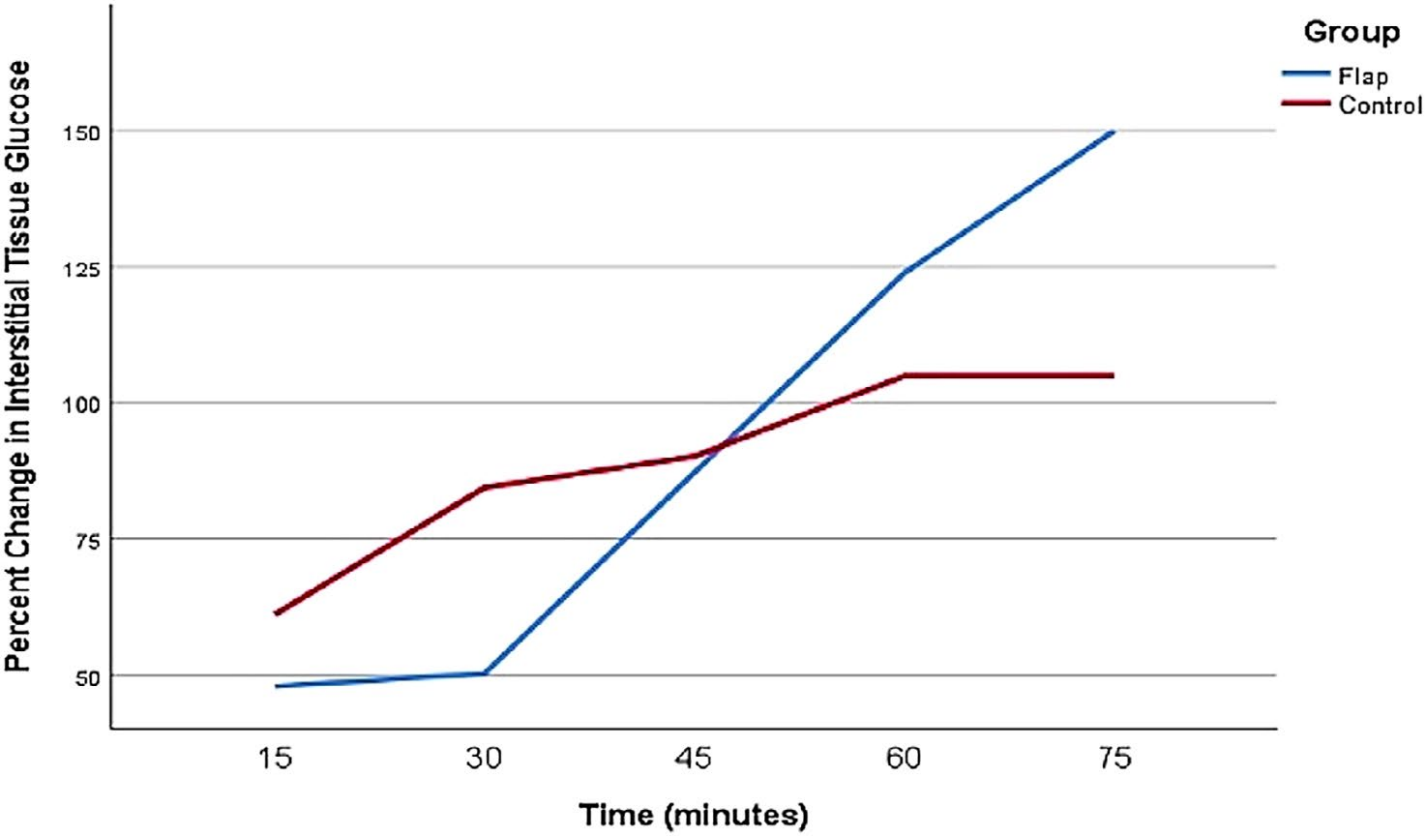
During the follow‐up after venous occlusion, an increase in glucose was observed in both the control device and the flap device. As seen in the graph, while the increase in the flap continues rapidly and is dependent on the occlusion time, the increase in the control device was self‐limiting.

During venous clamping, an additional interstitial glucose monitoring device was applied to the healthy tissue of the rabbit dorsum for control purposes. The measurements revealed an increase in control glucose levels, likely due to the systemic response to trauma (Wei et al. 2022). However, this increase was not continuous or time‐correlated with the rise observed in the flap; instead, it was a self‐limiting increase (Figure 11).

We also assessed the correlation between interstitial glucose levels and tissue decongestion following venous unclamping. Our measurements revealed a significant decrease in interstitial glucose levels after the clamp was released, suggesting that this reduction was attributable to the relaxation of the constricted interstitial tissue and the subsequent decrease in glucose concentration.

The magnitude of glucose increase may vary between each subject, but the percentage rise was similar. Therefore, we suggest that it is more informative to evaluate glucose increases by percentage change from baseline rather than by absolute increase. For this reason, we suggest that in clinical practice, tracking the percentage increase in glucose will be more informative rather than specifying a specific glucose level as a marker of venous congestion.

It is well established that arterial occlusion leads to a rapid decline in saturation and a loss of viability in a free flap (Keller 2009). Our hypothesis was that there would be a rapid decrease in the interstitial tissue glucose of the free flap when the sole source of nutrition was disrupted. The rapid decrease in glucose values measured at 15 min after arterial occlusion supports this hypothesis (Figure 7). Due to its operating mechanism, the glucometer permanently stops measuring after repeated measurements below 50 mg dL. In our preliminary studies, the rapid drop in glucose levels below 50 mg dL after arterial occlusion caused device failure after 45 min. Flap necrosis was also observed following irreversible arterial occlusions in our preliminary studies. Therefore, we decided to monitor arterial occlusions at 45‐min intervals. However, even though we prevented device failure, we continued to encounter irreversible arterial occlusions.

Another potential advantage of an interstitial glucose monitoring device is the ability for clinicians to differentiate between venous and arterial occlusion prior to surgical intervention. This capability reduces the exploration time required for re‐anastomosis and minimizes the duration the flap remains under traumatic conditions, thereby enhancing the likelihood of flap survival (Bui et al. 2007).

**FIGURE 12:**
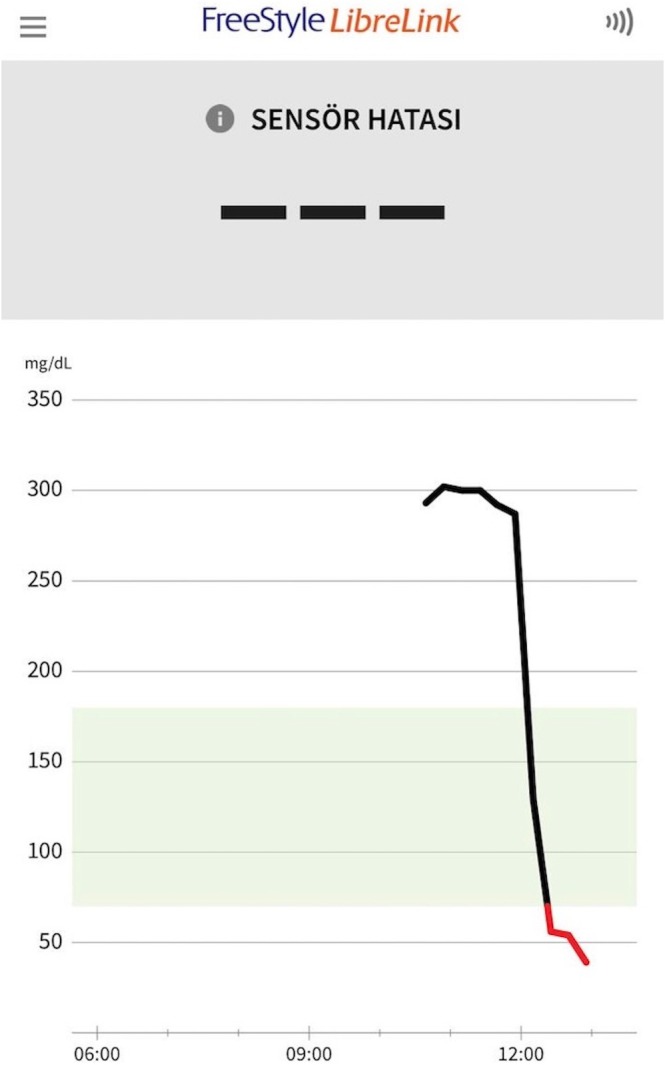
Screenshot taken from the device mobile application. Rapid glucose drop graph seen after arterial occlusion. Measurements could not be taken because the device gave a reading error at values below 50 mg/dL.

Several limitations of the present study warrant consideration. First, the free tissue transfer model used does not involve the creation of a microvascular anastomosis. Thrombosis in a vascular anastomosis is fundamentally different from vessel clamping, and this distinction may influence the rate at which interstitial glucose levels decline. Another limitation was our inability to monitor the return from arterial occlusion. Due to the operating mechanism of the device, it recommended using a new sensor after repeated measurement failures, which hindered arterial reperfusion monitoring (Figure 12). In addition, the thrombogenic nature of rabbits and the small vessel caliber may have contributed to irreversible arterial occlusion, resulting in undetected glucose levels. In clinical practice, flap reperfusion can be achieved after revision surgery for arterial occlusion, but because glucose measurement cannot be achieved for a long period, the sensor will stop measuring. Once reperfusion is achieved, a new sensor will need to be fitted to the flap.

## 5 Conclusion

In this experimental model, interstitial glucose monitoring allowed for the early detection of alterations consistent with reduced perfusion and gave real‐time quantitative data on flap metabolism. In controlled environments, such real‐time monitoring may help identify vascular impairment earlier and reduce the delay between onset and identification. Given the trial design and existing device constraints, routine clinical implementation of interstitial glucose monitoring cannot currently be advised, despite our data suggesting that it has potential as an additional tool for postoperative flap surveillance. To confirm these findings, establish clinically significant thresholds, and ascertain viability and dependability in human reconstructive microsurgery, more translational and clinical research is necessary.

## Author Contributions

All authors have seen and agreed to the submitted version of the paper, and bear responsibility for it.

## Funding

This work was funded by the Kocaeli University Scientific Research Projects Coordination Unit (TSA‐2024‐3697). The authors hereby declare that they have no other financial interest(s) in the material presented within.

## Ethics Statement

All procedures were approved by the Kocaeli University Animal Care and Use Institute Commission.

## Conflicts of Interest

The authors declare no conflicts of interest.

## Data Availability

The data that support the findings of this study are available from the corresponding author upon reasonable request.
